# Exposure to Sevoflurane Affects the Development of Parvalbumin Interneurons in the Main Olfactory Bulb in Mice

**DOI:** 10.3389/fnana.2016.00072

**Published:** 2016-06-24

**Authors:** Jing Yang, Jing Chen, Guohong Cai, Rui Lu, Tingting Sun, Tingting Luo, Shengxi Wu, Shucai Ling

**Affiliations:** ^1^Institute of Neuroscience and Anatomy, School of Medicine, Zhejiang UniversityHangzhou, China; ^2^Department of Anatomy and K.K. Leung Brain Research Center, Fourth Military Medical UniversityXi’an, China; ^3^Department of Neurobiology and Collaborative Innovation Centre for Brain Science, Fourth Military Medical UniversityXi’an, China; ^4^State Key Laboratory of Military Stomatology, Department of Anesthesiology, School of Stomatology, The Fourth Military Medical UniversityXi’an, China

**Keywords:** sevoflurane, olfactory bulb, PV, CB, CR

## Abstract

Sevoflurane is widely used in adult and pediatric patients during clinical surgeries. Although studies have shown that exposure to sevoflurane impairs solfactory memory after an operation, the neuropathological changes underlying this effect are not clear. This study detected the effect of sevoflurane exposure on the development of calcium-binding proteins-expressing interneurons in the main olfactory bulb (MOB). We exposed neonatal mice to 2% sevoflurane at two different developmental time points and found that exposing mice to sevoflurane at postnatal day (PD) 7 significantly decreased the expression of GAD67 and parvalbumin (PV) in the olfactory bulb (OB) but did not alter the expression of calretinin (CR) or calbindin D28k (CB). The number and dendritic morphology of PV-expressing interneurons in the MOB were impaired by exposure to sevoflurane at PD7. However, exposure to sevoflurane at PD10 had no effect on calcium-binding protein expression or the number and dendritic morphology of PV-expressing interneurons in the MOB. These results suggest that exposing neonatal mice to sevoflurane during a critical period of olfactory development affects the development of PV-expressing interneurons in the MOB.

## Introduction

Inhalation anesthetics are widely used in adult and pediatric patients during surgeries, and sevoflurane is one of the most frequently used inhalation anesthetics in infants and children because it has a low blood/gas ratio, low pungency and a rapid onset and recovery (Sakai et al., 2005; Edwards et al., 2010; Gibert et al., 2012). However, animal studies have shown that exposing animals to inhalation anesthetics during critical developmental periods can induce neuropathologic changes and impair cognitive functions (Culley et al., 2003, 2004; Jevtovic-Todorovic et al., 2003; Fredriksson et al., 2007; Rammes et al., 2009). Sevoflurane was shown to induce neuronal apoptosis and neuroinflammation and to inhibit neurogenesis in neonatal mice (Lu et al., 2010; Nie et al., 2013; Shen et al., 2013; Zhang et al., 2013). Exposing young rats to sevoflurane altered dendritic spine density during an important stage in synapse formation (Briner et al., 2010). These discoveries indicate that exposure to sevoflurane can affect the normal development of the brain. The olfactory system is vulnerable to volatile agents because its receptors are directly exposed to the outside environment. Although studies have shown that inhaling anesthetic drugs can lead to olfactory dysfunction (Adelman, 1995; Fukumoto et al., 2005), there are few reports showing the neuropathologic changes in olfactory system induced by sevoflurane exposure.

The olfactory system consists of the olfactory epithelium, the main olfactory bulb (MOB) and the olfactory cortex. The MOB is an important part of the olfactory system for olfactory functions. It has high plasticity because its neural network can be modified even by simple stimulation during an olfactory experience, such as exposure to an odorant (Buonviso et al., 1998; Buonviso and Chaput, 2000). The interneurons are the main population of neurons in the MOB, and they are even more sensitive to environmental stimulation because they have a prolonged developmental period and a highly dynamic nature, with continuous genesis occurring during the postnatal and adult periods (Altman, 1969; Lledo et al., 2004). Studies have shown that inducing early postnatal deprivation with uninaris occlusion can decrease the number of granule cells in the MOB and affect their maturation (Frazier-Cierpial and Brunjes, 1989; Brunjes, 1994). Olfactory enrichment can promote the survival of interneurons in the MOB during postnatal development (Mandairon et al., 2008). These discoveries demonstrated that sensory activity is essential for the normal development of interneurons in the MOB. No reports have shown whether inhaling anesthetic drugs affects neurons in the MOB in developing mice. In this study, we aimed to examine the effects of sevoflurane exposure on interneurons in the MOB of mice at different developmental stages.

Interneurons in the MOB use γ-aminobutyric acid (GABA) as their main neurotransmitter (Groh et al., 2004) and can be distinguished by their expression of different calcium-binding proteins, such as calretinin (CR), parvalbumin (PV), and calbindin D28k (CB; Parrish-Aungst et al., 2007). The CR-expressing neurons are scattered in the glomerular layer (GL), external plexiform layer (EPL) and granule cell layer (GCL) of the MOB, while CB-expressing neurons are mainly concentrated in the GL (Baimbridge et al., 1982; Hendrickson et al., 1991; Kiraly and Celio, 1991; Kosaka et al., 1995). PV-expressing neurons are mainly located in the EPL (Kosaka et al., 1987, 1994; Crespo et al., 1999; Hwang et al., 2003). These neurons form the main inhibitory circuits in the MOB, and changes in the expression of these calcium-binding proteins can define the functional characteristics of interneurons and impact olfactory functions. To investigate whether sevoflurane exposure during early postnatal life affects the later development of these neurons, we exposed neonatal mice to clinically relevant concentrations of sevoflurane (2%) for 6 h as used in the study of Yufune et al. (2016) at two distinct developmental stages in the MOB: postnatal days (PD) 7 and 10. We found that sevoflurane exposure at PD7 decreased the number of PV-expressing neurons and affected their dendritic development. CR- and CB-expressing neurons were not affected by sevoflurane exposure at either PD7 or PD10.

## Materials and Methods

### Experimental Animals

C57BL/6 mice were purchased from the Experimental Animal Center of the Fourth Military Medical University and individually housed with free access to food and water in a temperature- and humidity-controlled environment with a 12:12 h light/dark cycle. When the neonatal mice reached PD7 or PD10, the treatments were performed. All experimental procedures received prior approval from the Animal Use and Care Committee for Research and Education of the Fourth Military Medical University (Xi’an, China). Every effort was taken to minimize animal suffering and to reduce the number of animals used. A total of 12 liters containing 102 male offspring were used in this study.

### Anesthesia Treatment

When the pups reached PD7 or PD10, they were randomly divided into a sevoflurane-treated group and an air-treated control group. Mice in the sevoflurane-treated group were placed in a plastic container and continuously exposed to 2% sevoflurane for 6 h using air as a carrier with a gas flow of 2 liters/min. During sevoflurane exposure, the container was heated to 38°C. The control animals were exposed to air without sevoflurane. After 6 h of treatment, the pups were placed back into their maternal cages.

### Nissl Staining

For nissl staining, mice at PD14, PD28 and PD42 in each group were deeply anesthetized with sodium pentobarbital (50 mg/kg) and then perfused with 20 mL 0.01 M phosphate-buffered saline (PBS, pH = 7.4), followed by 100 mL 4% paraformaldehyde in 0.1 M phosphate buffer solution (PB, pH = 7.4). Then the Olfactory Bulbs (OB) were removed and post-fixed in the same fixative for 3 h and then cryoprotected for 24 h at 4°C in 0.1 M PB containing 30% sucrose. Coronal sections (30 μm) were cut in a freezing microtome (Leica CM1800, Heidelberg, Germany) at −20°C and collected in 0.01 M PBS. For staining, the sections were mounted on gelatin coated glass slides. When dried, the sections were defatted in 75% ethanol at 37°C overnight. Then the sections were stained for 10 min in 0.1% cresyl violet solution at RT, after rinsing with water, sections were incubated with 70% ethanol (3 s), 80% ethanol (3 s), 90% ethanol (3 s), 95% ethanol (3 s), absolute ethanol I (3 s) and absolute ethanol II (5 min) and then with xylene I (10 min) and xylene II (30 min). Sections were observed under an optical microscope after mounting with permount.

### Western Blot Analysis

When pups reached their ages, they were sacrificed, and the olfactory were rapidly removed. The tissue samples were homogenized using an ultrasonic wave (10 s, 3 times) in RIPA lysis buffer, which contained a cocktail of proteinase and phosphatase inhibitors (Roche). After centrifugation at 12,000 rpm for 15 min at 4°C, the protein-containing supernatants were collected. The protein concentrations were determined with a BCA-based kit (Pierce). Lysate samples were subjected to sodium dodecyl sulfate polyacrylamide gel electrophoresis; then, the proteins were transferred onto polyvinylidenedifluoride (PVDF) membranes (Bio-Rad). After blocked with 5% defatted milk in Tween/Tris-buffered saline (TBST) for 1 h at room temperature, the membranes were incubated with the primary antibody at 4°C overnight. The following primary antibodies were used: mouse monoclonal GAD67 (Chemicon^®^ 1:5000), mouse monoclonal CB (Sigma-Aldrich 1:1000), goat polyclonal CR (Abcam 1:2000), rabbit polyclonal PV (Abcam 1:1000) and mouse polyclonal β-actin (Sigma-Aldrich 1:5000). After washing in TBST, the membranes were incubated in a secondary antibody for 2 h at room temperature. All of the blots were detected by an enhanced chemiluminescence (ECL) detection system (Advansta). The scanned images were quantified with ImageJ (version 1.47) Software.

### Immunofluorescence Staining and Cell Counting

The mice atPD42 in each group were deeply anesthetized with a lethal dose of sodium pentobarbital (50 mg/kg of body weight) and then perfused with 0.01 M PBS (pH 7.4), followed by a 4% phosphate-buffered formalin in a 0.1 M phosphate buffer solution (pH 7.4). Coronal olfactory sections (30 μm) were cut in a freezing microtome (Leica CM1800, Heidelberg, Germany) at −20°C and collected in 0.01 M PBS. During the staining, the cryostated sections were washed in PBS three times, and following blocking in 10% normal donkey serum in PBS, the sections were incubated with primary antibodies overnight at 4°C. The following antibodies were used: mouse monoclonal CB (Sigma-Aldrich 1:500), goat polyclonal CR (Abcam 1:1000), and rabbit polyclonal PV (Abcam 1:500). After rinsing in PBS, the slides were incubated with secondary antibodies conjugated with Alexa Fluor 488 (Invitrogen/Life Technologies 1:500) for 2 h at room temperature and counterstained with 100 ng/ml DAPI. After rinsing in PBS, the sections were mounted on gelatin-coated glass slides and cover slipped in Flouromount G. The sections were observed and captured with a confocal laser scanning microscope (Olympus FV1000, Japan). Imaging-Pro-Plus (LEIKA DMLB) was used to perform quantitative analysis of the positive immunostained cells numbers. Every fourth coronal section through the OB was collected, and a total of five sections from each mouse were used for quantification. The number of immunostained cells in each field was counted at a higher magnification (200×). Three random fields were captured in each section, and the mean number of immunostained neurons per view in the three views was included as the data for each section. The final average number of immunostained neurons per vision in all sections was included as the data for each sample.

### Statistic Analysis

All data were presented as Mean ± SD. The statistical analysis was performed with GraphPad Prism 5.0 Software (GraphPad Software) and comparisons of the means of two groups were performed using the Student *t* test. Statistical significance was inferred at *P* < 0.05.

## Results

### Effects of Sevoflurane Exposure at PD7 and 10 on the Laminar Organization of the MOB

**Figure 1:**
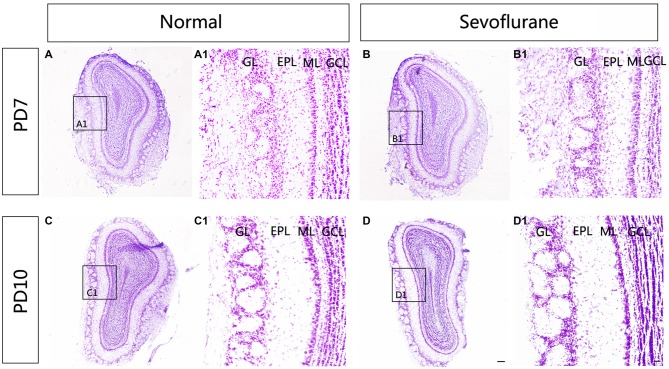
**The effect of exposure to sevoflurane at postnatal days 7 (PD7) and 10 (PD10) on the laminar organization of main olfactory bulb (MOB).** No significant change in the laminar organization of the MOB was observed between control mice **(A)** and mice exposed to sevoflurane at PD7 **(B)**. Compared to control MOBs **(C)**, the laminar organization of the MOB in mice that were treated with sevoflurane at PD10 **(D)** was unchanged. **(A1–D1)** High magnification images showing the structure of the MOB. Scale bars = 500 μm in **(D)** (applies to **A–D**) and 100 μm in **(D1)** (applies to **A1–D1**).

To explore the effect of sevoflurane exposure on the overall morphology of the MOB, Nissl staining was used to show the structure of the MOB. A distinct laminar organization can be seen in the MOB, with intact glomeruli and clear layers. Following sevoflurane exposure at PD7 and PD10, the organization of the MOB was the same as that in control mice (Figure 1).

### Changes in the Expression of GAD67 and Calcium-Binding Proteins in the OB of Mice Exposed to Sevoflurane at PD7

**Figure 2:**
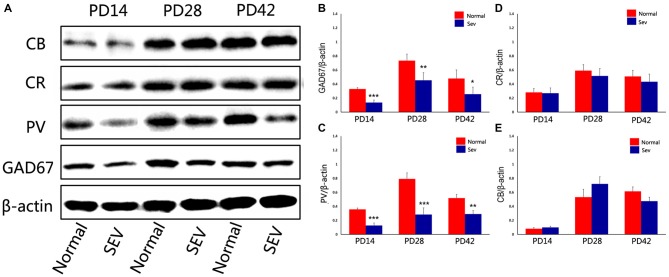
**The effect of exposure to sevoflurane at PD7 on GAD67 and calcium-binding protein expression. (A)** Representative autoradiogram showing the expression of GAD67 and calcium-binding proteins in the MOB at PD14, 28 and 42. Normal: control group. SEV: sevoflurane-treated group. Quantitative analysis of the results of western blot analysis to determine the expression of GAD67 **(B)**, parvalbumin (PV) **(C)**, calretinin (CR) **(D)**, and calbindin D28k (CB) **(E)** Values are expressed as the Mean ± SD. ***P < 0.001, **P < 0.01, and *P < 0.05.

To assess the effect of sevoflurane exposure at PD7 on interneurons in the OB at different developmental stages, we first analyzed GAD67 expression in the OB in the sevoflurane-treated mice and control mice at PD14, 28 and 42 using western blot analysis. Compared to the control group, the expression of GAD67 was significantly decreased at PD14, 28 and 42 in the OB in the sevoflurane-treated mice. The significance of this difference was decreased as the age of the mice increased (Figures 2A,B; *P* = 0.0001 at PD14, *P* = 0.000477 at PD28, and *P* = 0.00156 at PD42). To explore which subtypes of interneurons were affected, the expression of calcium-binding proteins was analyzed using western blot analysis. Compared to control mice, sevoflurane exposure at PD7 significantly decreased the expression of PV in the OB of mice when analyzed at PD14, 28 and 42 (Figures 2A,C; *P* < 0.001 at PD14 and 28, *P* < 0.01 at PD42). However, there were no significant differences in the expression of CB and CR between the sevoflurane-treated mice and the control mice (Figures 2A,D,E).

### Effects of Sevoflurane Exposure at PD7 on the Number of Calcium-Binding Protein-Expressing Neurons in the MOB

**Figure 3:**
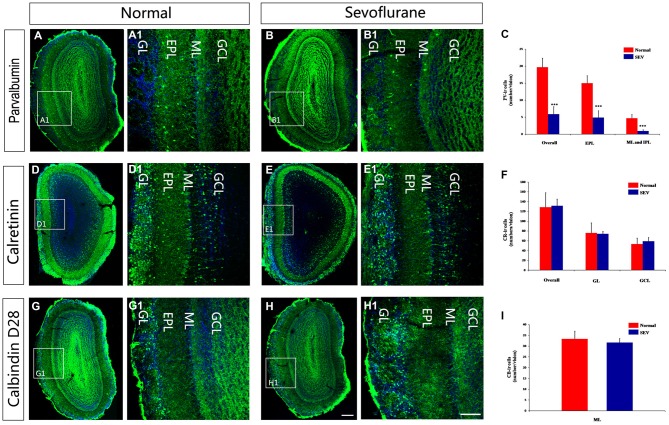
**The effect of exposure to sevoflurane at PD7 on the number of calcium-binding protein-expressing neurons in the MOB.** Control mice **(A)** had more PV interneurons in the MOB than the sevoflurane-treated mice **(B). (A1,B1)** High magnification images of PV interneurons in the MOB. The density of CR interneurons in the MOB of mice exposed to sevoflurane at PD7 **(D)** was unaffected compared to the control mice **(E). (D1,E1)** High magnification images of CR interneurons in the MOB. The density of CB interneurons in the MOB of mice exposed to sevoflurane at PD7 **(G)** was unaffected compared to the control mice **(H)**. **(G1,H1)** High magnification images of CB interneurons in the MOB. Normal: control group. SEV: sevoflurane-treated group. Quantification analysis of the number of PV-ir neurons **(C)**, CR-ir neurons **(F)** and CB-ir neurons **(I)** in different layers of MOB. Scale bars = 100 μm (applies to **A–H,A1–H1**). Values are expressed as the Mean ± SD. ***P < 0.001.

To determine whether the observed molecular changes represented alterations at the cellular level, immunofluorescence staining was performed to display calcium-binding proteins in immunoreactive (ir) interneurons. The pattern of distribution for PV-ir, CR-ir and CB-ir interneurons were not qualitatively different between sevoflurane-treated mice and control mice. The soma of the PV-ir interneurons were mainly located in the EPL and few PV-ir cells located in ML and IPL. The CB-ir interneurons were periglomerular interneurons, and the CR-ir interneurons were observed in all layers of the MOB, with the highest packing density in the GL (Figures 3A–F). Quantitative estimations of PV-ir interneuronal density in the MOB in PD42 mice revealed that sevoflurane exposure at PD7 significantly decreased the number of PV-ir neurons not only in EPL but also in the ML and IPL (Figure 3G, *P* < 0.001 respectively). However, sevoflurane exposure at PD7 did not induce a striking change in the density of CR-ir and CB-ir interneurons compared to their numbers in the controls (Figures 3H,I).

### Morphological Alterations in PV-expressing Interneurons in the MOB of Mice Exposed to Sevoflurane at PD7

**Figure 4:**
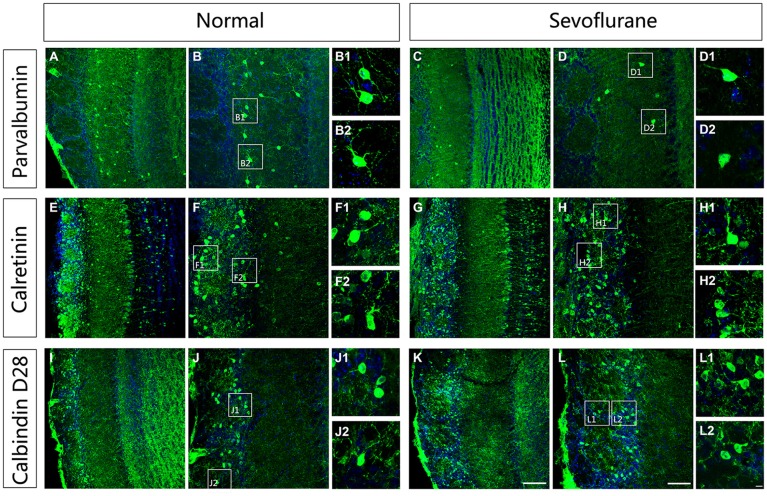
**The effect of exposure to sevoflurane at PD7 on the dendritic architecture of calcium-binding protein-expressing neurons in the MOB.** Compared to the control group **(A,B)**, the dendritic morphologies of PV interneurons were impaired by exposure to sevoflurane at PD7 **(C,D). (B1,B2,D1,D2)** High magnification images of PV interneurons in the MOB. Compared to the control group **(E,F)**, the dendritic morphologies of CR interneurons were not impaired by exposure to sevoflurane at PD7 **(G,H). (F1,F2,H1,H2)** High magnification images of CR interneurons in the MOB. Compared to the control group **(I,J)**, the dendritic morphologies of CB interneurons were not impaired by exposure to sevoflurane at PD7 **(K,L). (J1,J2,L1,L2)** High magnification images of CR interneurons in the MOB. Scale bars = 100 μm in **(K)** (applies to **A,C,E,G,I,K**), 50 μm in **(L)** (applies to **B,D,F,H,J,L**), and 5 μm in **(L2)** (applies to **B1,B2,D1,D2,F1,F2,H1,H2,J1,J2,L1,L2**).

To further evaluate the effect of sevoflurane on neuronal morphologies, immunofluorescence staining was used to display the dendritic arbor architecture of these interneurons in control and sevoflurane-exposed mice at PD42. The results showed that the PV-ir interneurons in the MOB in control mice were multipolar neurons with extensive and clear dendrite complexities with visible secondary and tertiary dendrites (Figures 4A–B2). In the sevoflurane-treated mice, the dendritic branches of PV-ir interneurons were significantly decreased, and some of these neurons had no dendrites (Figures 4D–D2). Unlike in the PV-expressing neurons, the dendrites of the CR-ir and CB-ir interneurons were not affected by sevoflurane exposure at PD7 (Figures 4E–L).

### Effects of Sevoflurane Exposure at PD10 on the Expression of GAD67 and Calcium-Binding Proteins in the OB in Mice

**Figure 5:**
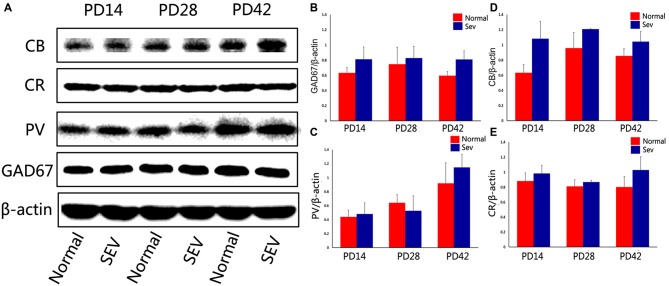
**The effect of exposure to sevoflurane at PD10 on GAD67 and calcium-binding protein expression. (A)** Representative autoradiogram of GAD67 and calcium-binding protein expression in the MOB at PD14, 28 and 42. Normal: control group. SEV: sevoflurane-treated group. Quantitative analysis of western blot results showing the expression of GAD67 **(B)**, PV **(C)**, CB **(D)**, and CR **(E)**. Values are expressed as the Mean ± SD.

To further explore the effect of sevoflurane exposure on phases of OB interneuron development, mice were subjected to sevoflurane exposure at PD10, and the expression of GAD67 and calcium-binding proteinswas detected using western blot analysis. At PD14, 28 and 42, there were no significant differences in the expression of GAD67, PV, CB, and CR in the OB between control mice and sevoflurane-exposed mice (Figure 5, *P* > 0.05).

### Effects of Sevoflurane Exposure at PD10 on the Numbers and Morphologies of PV-expressing interneurons

**Figure 6:**
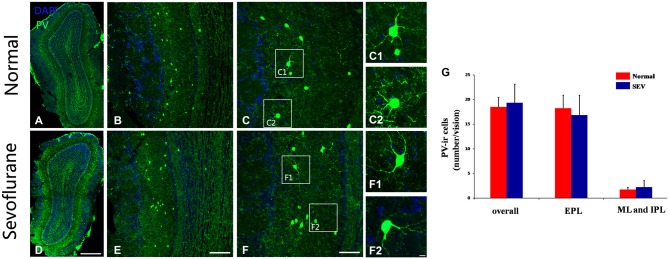
**The effect of exposure to sevoflurane at PD10 on the number and dendritic morphology of PV interneurons.** Compared to the control group **(A–C)**, the number and dendritic morphologies of PV interneurons were not impaired by exposure to sevoflurane at PD10 **(D–F)**. **(C1,C2,F1,F2)** High magnification images of PV interneurons in the MOB. **(G)** Quantification analysis of the number of PV-ir neurons in different layers of MOB. Scale bars = 200 μm in **(D)** (applies to **A,D**), 100 μm in **(E)** (applies to **B,E**), 50 μm in **(F)** (applies to **C,F**), and 5 μm in **(F2)** (applies to **C1,C2,F1,F2**). Values are expressed as the Mean ± SD.

To further confirm the effects of sevoflurane exposure at PD10 on PV-expressing interneurons at the cellular level, immunofluorescence staining was used to detect the numbers and morphologies of PV-ir interneurons in the MOB of control and sevoflurane-treated mice at PD42. No difference was observed in the number of PV-ir interneurons not only in EPL but also in the ML and IPL of the MOB between control mice and sevoflurane-treated mice (Figures 6A,B,D,E,G, *P* > 0.05). The dendrites of the PV-ir neurons in the MOB were clear and ramulose in both the control mice and the sevoflurane-exposed mice (Figures 6C–C2,F–F2). This indicated that sevoflurane exposure at PD10 had no effect on the PV-expressing interneurons of the MOB.

## Discussion

In the present study, by exposing neonatal mice to sevoflurane, we explored the effects of sevoflurane on interneurons in the OB of developing mice. First, we found that the laminar organization of the MOB was not adversely affected by sevoflurane exposure at either PD7 or PD10. Second, sevoflurane exposure at PD7 affected the survival and dendritic development of PV-expressing interneurons in the MOB. Finally, sevoflurane exposure at PD10 had no effect on calcium-binding protein-expressing interneurons in the MOB.

Sevoflurane is one of the most commonly used anesthetics in neonatal and pediatric patients (Lerman et al., 1994). A report showed that the perception of smells was altered during inhalational induction in children (Verschuur et al., 2005). To explore whether sevoflurane exposure during the early neonatal period induces neuropathological changes in the olfactory system for olfactory dysfunctions, the OB was chosen because it is the first relay station for the transmission of olfactory information and because it is highly plastic. Recent studies have shown that there is a critical developmental period for the mammalian olfactory system (Ma et al., 2014; Tsai and Barnea, 2014). The discoveries of Tsai and Barnea (2014) and Ma et al. (2014) demonstrated that the first postnatal week is the critical period for glomerular map organization in the OB. Meanwhile previous study demonstrated that immature brains are, in general, the most vulnerable to anesthesia at PD7 (Yon et al., 2005). Considering this critical period, we chose two developmental time points that fell within the early postnatal period. We first detected the effects of sevoflurane exposure at PD7 and PD10 on the laminar organization of the MOB at different developmental stages and found that sevoflurane exposure during the early postnatal period did not impair the laminar architecture or volume of the MOB in mice (Figure 1). The clear glomerular structures of the MOB in mice are developed around PD4, and their construction can be affected by olfactory activities (Royal and Key, 1999; Potter et al., 2001; Zou et al., 2004). In some diseases that are associated with olfactory malfunctions, such as schizophrenia, patients display a smaller MOB (Turetsky et al., 2000, 2003). Here, the intact laminar organization of the MOB may indicate that basic olfactory functions are preserved after sevoflurane exposure.

A previous report demonstrated that olfactory acuity was intact in patients after anesthesia with sevoflurane, whereas olfactory memory was impaired (Kostopanagiotou et al., 2011). Although the mechanisms underlying this effect are not understood, previous studies have mainly focused on the effects of sevoflurane on GABAergic circuits (Bein et al., 2008; Michel and Constantin, 2009; Kotani and Akaike, 2013). In the MOB, the ratio of local GABAergic interneurons to excitatory neurons is 100:1(Groh et al., 2004). These interneurons comprise the local circuitry in the MOB. In our study, we found the expression of GAD67 was decreased in the OB in mice after sevoflurane exposure at PD7. The most dramatic decrease in its expression was observed at 1 week after sevoflurane exposure. This effect was reduced as the age of the mice increased (Figures 2A,B). In the MOB, most interneurons express GAD67, and GAD67 is expressed in the soma and puncta of neurons, where it is responsible for maintaining baseline levels of GABA (Tsunekawa et al., 2005). The decreased expression of GAD67 might indicate changes in the numbers or functions of interneurons in the MOB. Different subtypes of GABAergic interneurons can be classified by molecular makers, such as CR, PV and CB (Parrish-Aungst et al., 2007). These calcium-binding protein-expressing neurons are the main populations of interneurons in the MOB. The expression of PV was dramatically reduced in the OB in the animals exposed to sevoflurane at PD7, and this effect was not reversed as the mice increased in age (Figures 2A,C). Cell counting was performed to determine the number of PV-ir interneurons in the MOB after sevoflurane inhalation at PD7. The results showed that the number of PV-containing interneurons was significantly decreased of MOB in the sevoflurane-treated mice at later ages (Figures 3A,A1,B,B1,G). However, no changes were observed in the expression of PV or the number of PV-ir neurons in the mice exposed to sevoflurane at PD10 (Figures 5A–C). It was widely accepted that sevoflurane exposure induced neuronal apoptosis in developing brains (Satomoto et al., 2009; Lu et al., 2010; Fang et al., 2012). A previous study demonstrated that immature brains are, in general, the most vulnerable to anesthesia-induced neuro-apoptosis at PD7 (Yon et al., 2005). Therefore, we concluded that sevoflurane exposure during this critical period of olfactory development affected the survival of PV-expressing interneurons in the MOB.

In humans and rodents, postnatal development is also an important period for the precise formation of neuronal circuits in the central nervous system (Petit et al., 1988; Brown et al., 1997; De Felipe et al., 1997). This postnatal period of immense growth is marked by synaptogenesis, which involves dendritic branching and the formation of synapses (Micheva and Beaulieu, 1996). The study of De Felipe et al. (1997) showed that in the rodent cortex inhibitory synaptogenesis begins at early time, and they find both asymmetrical and symmetrical synapses were present in all layers from P4. Meanwhile, discovery from the development of the adult new-born interneurons showed that GABAergic and glutamatergic synaptic contacts increased at 7 dpi (Panzanelli et al., 2009). Previous studies have shown that exposure to anesthesia during this phase can affect the normal development of spines and synapses (Briner et al., 2010; Lunardi et al., 2010; Amrock et al., 2015). In our study, we found that exposure to sevoflurane at PD7 decreased dendritic branches in PV-ir interneurons in the MOB (Figures 4C,D–D2), whereas sevoflurane exposure at PD10 did not affect dendritic architecture (Figures 6A–F). These results indicate that exposure to sevoflurane during peak synaptogenesis affects the normal development of dendrites in PV neurons in the MOB.

Interneurons in the OB are a heterogeneous population that is produced beginning in embryogenesis and continuing through adulthood. The development of different calcium-binding protein-expressing neurons occurs in a spatially and temporally regulated manner (Stenman et al., 2003; Waclaw et al., 2006; Young et al., 2007; Batista-Brito et al., 2008; Li et al., 2011). Different interneurons are preferentially produced at different ages. PV-expressing interneurons are produced from late embryogenesis to early postnatal stages (E17 to P3). After P5, there is no new production of PV-expressing neurons in the OB (Li et al., 2011). Although a previous study showed that after ischemic lesion, progenitors in the SVZ can migrate to the lesion site and differentiate into mature PV-containing neurons, there is no evidence that a lesion in the OB can induce the regeneration of PV interneurons (Teramoto et al., 2003). This may explain why the loss of PV-expressing interneurons that was induced by sevoflurane exposure at PD7 was not compensated when the mice matured. Unlike PV interneurons, CB- and CR-expressing interneurons can be generated throughout postnatal stages, and CR-expressing interneurons make up the largest proportion of neurons that are born in adult mice (Batista-Brito et al., 2008; Li et al., 2011).In our study, we found that sevoflurane exposure PD7 or PD10 did not affect the number of CB- and CR-expressing interneurons in the MOB (Figures 2A,B,E, 5A,D,E). Previous reports showed that exposing PD4-6 neonatal rats to 1.8% sevoflurane for 6 h promoted hippocampal neurogenesis (Chen et al., 2015). Therefore, we assumed that sevoflurane exposure might promote neurogenesis in the OB, and that this may have contributed to fact that there was no change in the number of CB- and CR-expressing interneurons. However, further studies should be performed to test this assumption.

In conclusion, this study is the first to describe the effect of sevoflurane exposure at two different developmental time points on the development of interneurons in the MOB. The results suggest that in neonatal mice, exposure to 2% sevoflurane of at PD7 can affect the survival and dendritic development of PV-expressing interneurons in the MOB, but it does not affect CB- and CR-expressing interneurons in the MOB. These findings may lay a morphological foundation for studies aimed at determining the effects of sevoflurane exposure on olfactory functions.

## Author Contributions

JY designed the experiments and drafted the manuscript. JC and GC participated in the study design and coordination. JY performed the experiments on immunofluorescence and western blotting and analyzed these data with TS. RL performed experiment on animal treatment with anesthesia. TL performed experiment on Nissl staining. SL and SW provided the financial and administrative support for this project. All authors read and approved the final manuscript.

## Conflict of Interest Statement

The authors declare that the research was conducted in the absence of any commercial or financial relationships that could be construed as a potential conflict of interest. The handling Editor declared a shared affiliation, though no other collaboration, with several of the authors JC, GC, RL, TL and SW and states that the process nevertheless met the standards of a fair and objective review.

## Abbreviations

CB: calbindinD28k
CR: calretinin
ECL: chemiluminescence
EPL: external plexiform layer
GABA: γ-aminobutyric acid
GCL: glomerular cell layer
GL: glomerular layer
MOB: main olfactory bulb
PD: postnatal days
PV: parvalbumin
PVDF: polyvinylidenedifluoride.
